# Impact of the Family Environment on the Frequency of Animal-Based Product Consumption in School-Aged Children in Central Poland

**DOI:** 10.3390/nu15122781

**Published:** 2023-06-17

**Authors:** Ewelina Pałkowska-Goździk, Katarzyna Zadka, Danuta Rosołowska-Huszcz

**Affiliations:** Department of Dietetics, Institute of Human Nutrition Sciences, Warsaw University of Life Sciences (SGGW-WULS), 159C Nowoursynowska Street, 02-776 Warsaw, Poland; katarzynazadka86@gmail.com (K.Z.);

**Keywords:** animal-based products, frequency of food consumption, determinants, nutritional survey, children

## Abstract

Animal-sourced foods (ASFs) have a high nutritional value, which makes them important elements of young people’s diets. Several environmental factors might affect the dietary habits of children and adolescents, and their identification seems to be a principal reason to maintain their healthy eating practice. Thus, we aimed to investigate selected environmental factors (a place of residence, net income, mother’s education level, number of siblings, and mother’s BMI), which may be linked to the consumption frequency of ASFs among school-aged children. In total, 892 mothers of primary school children aged 7–14 years from central Poland took part in the anonymous and voluntary survey. The frequency of meat and meat product consumption was affected by the mother’s education level, place of residence, and net income. Generally, meat was eaten more often by the city children (G = 0.178, *p* < 0.01) of better-educated mothers (G = 0.268, *p* < 0.001) and higher-income families (G = 0.209, *p* < 0.001). A higher level of education was linked to more frequent fish consumption but only in the younger group (G = 0.130, *p* < 0.05). The frequency of egg intake was positively associated with the maternal level of education (G = 0.185, *p* < 0.001), children’s gender (girls > boys, G = 0.123, *p* < 0.05), and place of residence (city > village, G = 0.214, *p* < 0.001). In turn, the frequency of milk and dairy intake was related only to the place of residence (village > city, G = 0.97, *p* <0.05). It can be concluded that the mother’s level of education is a key factor linked to the selected children’s dietary habits. Thus, we believe that successful health education programs designed for young people should include the maternal capacity to interpret and adapt information into daily practice.

## 1. Introduction

A healthy diet includes individually balanced energy intake and consumption of a wide range of nutrient-dense foods, starting with vegetables, fruits, whole-grain cereal products, low-fat dairy products, and a variety of protein foods. Animal-based products—particularly fish, meat, eggs, and milk—have high nutritional value [1,2]. They provide a blend of highly bioavailable nutrients and bioactive components, which are necessary in the diets of children and adolescents to support healthy growth and development, including essential amino acids, essential fatty acids, zinc, calcium, heme-iron, vitamin A, and B vitamins [1,2]. 

Meat and meat products are a major component of the Western diet. Growing consumer concerns over health, environmental sustainability, and animal welfare affect meat consumption habits [3]. Today, there is a general dietary recommendation set forward by The World Health Organization [4] and by a number of national health advice bodies (presented also in Poland’s food guidelines as Plate of Healthy Eating) to eat less red and processed meat in favor of fish, poultry, eggs, legume seeds, and nuts [5]. 

The level of fish consumption is diverse worldwide and influenced by several factors [6], but data indicate insufficient compliance with fish intake recommendations in both adult [7,8] and child populations [9,10], which might subsequently increase the risk of low intake of omega-3 polyunsaturated fatty acids (PUFAs), mainly docosahexaenoic acid (DHA). 

Data from The Hellenic National Nutrition and Health Survey (HNNHS) showed that the majority of the study population (95%) consumed fewer than five eggs per week [10]; in the Polish arm of the Prospective Urban Rural Epidemiological Study, a similar level of consumption was observed—the majority of female (91.2%) and male (82.3%) study participants ate no more than four eggs weekly [11]. There is evidence that egg consumption is associated with better nutritional status in children [12], greater recumbent length, higher protein, choline, lutein/zeaxanthin, vitamin B12, and selenium intake compared to infants (6–24 months of age) not consuming them [13]. Similarly, the National Health and Nutrition Examination Survey (NHANES) revealed that in children and adolescents (2–18 years), egg consumption was linked to a greater intake of protein, DHA, α-linolenic acid, potassium, selenium, choline, lutein + zeaxanthin, vitamins B2, E, and D, and lower sugar intake versus dietary patterns with no eggs added [14]. 

Milk and milk products are frequent diet components of the Polish population. In a study involving the Upper Silesian population, most of the respondents (70.5%) were shown to drink milk several times a week, and there were no people who denied consuming milk. Fermented milk drinks (natural and fruit) and cheese (the main indication was cottage cheese) were also consumed several times per week (2–6/week) (55.0%, 58.2%, and 60%, respectively) [15]. According to the results of the survey conducted in schoolchildren from elementary schools, 48% of them consumed milk and dairy products every day and 42% several times per week [16]. 

The food consumption pattern is a key health-shaping factor. In turn, consumer behavior in the process of purchasing, and food consumption is complex and influenced by several factors [17,18]. Among the determinants of food choices, not only dietary components but also personal, cultural, social, physiological, and psychological pressures should be mentioned [17,18]. During childhood and early adolescence, nutritional habits/nutritional awareness are mainly affected by parental BMI, food preferences, educational level, socioeconomic position, and the availability of food at home [19]. Parents play an important role in health-promoting and environmentally friendly practices [20].

The present study was, therefore, designed to determine the relationship of selected environmental factors, such as the place of residence, economic status (net income per family), parental education level, number of children, and the respondent BMI (mother), on the frequency of animal-based food consumption (i.e., meat and meat products, fish, eggs, milk, and dairy) by school-age children in central Poland.

## 2. Materials and Methods

### 2.1. Study Population and Survey Instrument

Study subjects and enrollment process were described previously in detail [21]. Briefly, we present a secondary analysis of data obtained in an anonymous voluntary survey completed by parents/legal guardians of children born in 2003–2010 and attending grades 1–6 of primary school (7–14 years old). The study involved parents whose child: was 7–14 years old on the day of the study, did not suffer from chronic diseases requiring dietary modification other than a food allergy, did not suffer from any diseases that made it impossible to eat independently, and was not fed enterally or parenterally. Failure to meet any of the inclusion criteria or incorrect completion of the questionnaire resulted in exclusion from further analyses. In general, we received 930 completed questionnaires; and due to missing data or other formal irregularities, 38 questionnaires had to be rejected, and data from 892 documents were used for further analysis.

Data were collected from 8 randomly selected schools placed in a city of 100,000 inhabitants (4 schools) and villages (with no more than 2000 residents at least 50 km away from the nearest urban center of this size) (4 schools) from central Poland between September 2016 and March 2017. We used a semi-structured questionnaire based on the tool the Dietary Habits and Nutrition Beliefs Questionnaire (KomPAN), validated by the Committee of Human Nutrition, The Polish Academy of Sciences [22], filled in only by the mothers during a parental meeting at their children’s school. Therefore, 100% of the respondents were mothers.

The form consisted mainly of closed questions concerning lifestyle, with particular emphasis on children’s dietary habits, frequency of consumption of selected foods, and the level of physical activity, which was discussed earlier [21]. To assess the frequency of consumption of selected product groups by children of mothers involved in the survey, each of the single-choice questions had the following answers: never, 1–3 times a month, once a week, several times a week, once a day, and several times a day. There were questions regarding children’s special nutritional needs, including vegetarian and vegan diet and food allergies. We also asked about the place of residence, economic status (net income per family), parental education level, number of children, weight, and height of both the respondent and the child. Based on these values, the mother’s BMI was calculated. We selected above-mentioned factors to assess their relationship with the frequency of animal product consumption. As animal products, we considered: meat, meat products (cold cuts, sausages, etc.), fish, dairy products, and eggs. Seafood was excluded due to the lack of people declaring their consumption. Honey was only occasionally consumed with tea by some respondents (4 of 892); hence, it was also omitted in the final analyses.

The Ethics Committee of the Faculty of Human Nutrition and Consumer Sciences of the Warsaw University of Life Sciences (SGGW-WULS) in Warsaw, Poland, approved the study (No. 11/2017). We obtained written informed consent from all participants before enrolment. 

### 2.2. Statistical Analyses

To verify whether the association of these factors changes with the child’s age, we compared their effects in two age groups: in the younger 7–10-year-old children (N = 510) and in the older (11–14-year-old) ones (N = 382). We also checked if the consumption of animal products was related to the consumption of other product groups, including vegetables, fruits, and whole-grain products. For all statistical analyses, PAWS Statistics 18 software (SPSS Inc., Hong Kong, China) was used. The Goodman and Kruskal’s gamma (G) was run to determine the association between the analyzed parameters. Statistical significance was estimated at *p* ≤ 0.05.

## 3. Results

A total of 892 mothers of school-aged children from eight schools (four village and four city schools) participated in the study (mothers accounted for 100% of the respondents). 

The characteristics of the participants were presented earlier [21] (Table 1).

### 3.1. The Frequency of Meat and Meat Product Consumption 

**Figure 1:**
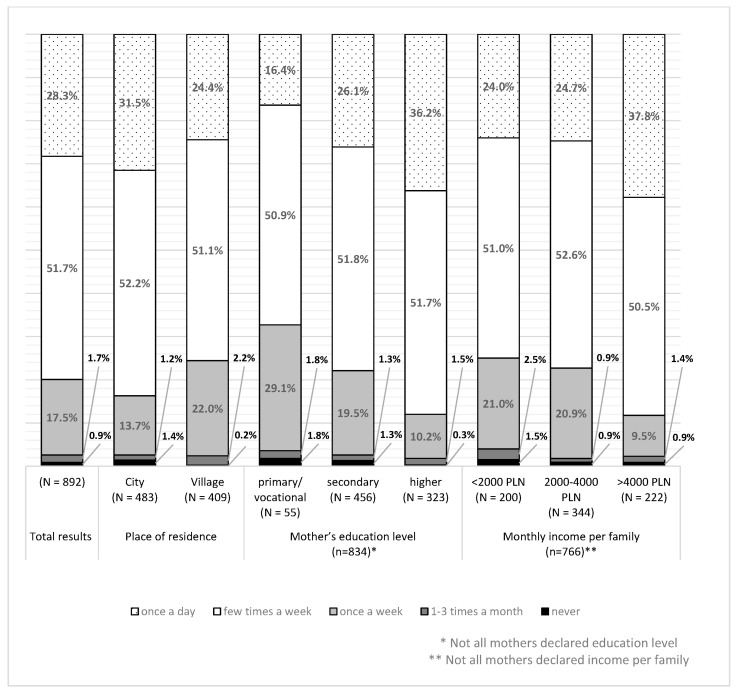
The frequency of meat and meat product consumption as dependent on the place of residence, the mother’s education level, and monthly income.

Based on the mother’s declarations, the highest percentage of children ate meat and meat products a few times a week or once a day (Figure 1). 

The frequency of meat consumption was related to the place of residence, the mother’s level of education, and net income per family. Children living in the city ate meat more often than those in the countryside (G = 0.178, *p* < 0.01). Higher levels of education and higher income were also associated with more frequent meat consumption (G = 0.268, *p* < 0.001 and G = 0.209, *p* < 0.001, respectively). 

There were no relationships between the frequency of meat consumption and children’s age, gender, body mass, and the number of siblings. The BMI value of the mothers also did not affect the frequency of meat consumption by their children. 

In the younger group (7–10 years), the frequency of meat consumption was higher in children living in the city (G = 0.217, *p* < 0.01), of better-educated mothers (G = 0.321, *p* < 0.001), and with higher income (0.241, *p* < 0.001). In the group of older children (11–14 years), the frequency of meat consumption was higher in children of better-educated mothers (G = 0.176, *p* < 0.05) and with higher income (0.159, *p* < 0.05). 

In the group of 892 mothers who completed questionnaires, 8 women indicated that their child did not eat meat or meat products at all, but only 3 mothers declared that it was connected with a vegetarian diet. Despite avoiding meat consumption, their diet included fish, eggs, and dairy products. Thus, based on those declarations, they can be considered lacto-ovo-vegetarians. 

Chicken was the most commonly eaten meat among children. Pork came second, followed by turkey. The most-often-consumed meat product was ham, followed by hotdogs and sausages. Home-made cold cuts were the least frequently eaten (Table 2 and Table 3).

### 3.2. The Frequency of Fish Consumption 

The frequency of fish consumption was described earlier [21]. There were no statistically significant associations between the frequency of fish consumption in the general population and the factors under examination. 

In the younger group, only a higher frequency of fish consumption was connected with a higher level of mother’s education (G = 0.130, *p* < 0.05). 

### 3.3. The Frequency of Egg Consumption 

**Figure 2:**
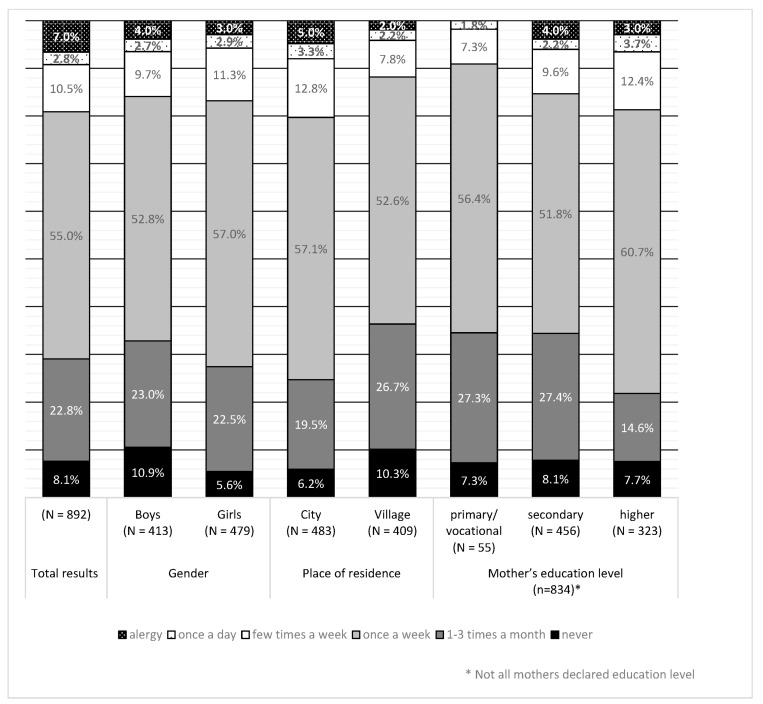
Frequency of egg consumption according to gender, place of residence, and mother’s education level.

The mothers most often declared that their children consumed eggs once a week or 1–3 times per month (Figure 2).

The frequency of egg consumption was associated with the children’s gender, place of residence, and the mothers’ education level. Eggs in the diet appeared more often in girls (G = 0.123, *p* < 0.05), urban residents (G = 0.214, *p* < 0.001), and in children of mothers with a higher level of education (G = 0.185, *p* < 0.001).

In both age groups, place of residence and mother’s education affected the frequency of egg consumption. A higher frequency of eggs in diets was observed in children living in a city (younger—G = 0.197, *p* < 0.05 and older—G = 0.264, *p* = 0.01) and children of mothers with a better education level (younger—G = 0.199, *p* <0.05 and older—G = 0.190, *p* < 0.05). 

Regardless of the studied factors, boiled eggs were eaten more often than fried ones.

### 3.4. The Frequency of Dairy Product Consumption

The frequency of dairy product consumption was presented previously [21]. In the general population, the place of residence was the only factor that was significantly associated with the frequency of dairy product consumption. Village children are more likely to eat dairy products (G= −0.186, *p* < 0.01), as stated previously [21]. 

In turn, in younger children, we observed the effect of gender, the number of siblings, and the place of residence as well. Girls consumed dairy products more often than boys (G = −0.187, *p* < 0.05), and the greater number of siblings (G = 0.150, *p* < 0.05) and living in the countryside favored more frequent consumption of dairy products (G = −0.289, *p* < 0.001). 

In the group of children aged 11–14, an influence of the examined factors was not found.

The associations between the frequency of animal-sourced food, vegetable, and whole-grain product intake are presented in Table 4. 

## 4. Discussion

While in previous papers, we investigated the relationship between environmental factors and children’s health behaviors contributing to the occurrence of diet-related diseases [21], here, we focused on environmental factors affecting the frequency of animal-based food consumption in school-aged children.

The frequency of meat and meat product consumption was the highest among the evaluated animal-based foods. Other results from nutritional surveys, including children and adolescents from elementary and secondary schools in Poland, also revealed frequent meat consumption. Similar to our study, it was demonstrated that white meat was one of the most popular types of products consumed for lunch by junior high and secondary school adolescents (86.9% and 87,4%, respectively), and further down the list, there was red meat (47.3% and 46.6%) [16]. Our findings are also compatible with data obtained in the survey including 90 parents of 5–8 years old children from Germany; 44% of children ate meat 2–3 times per week, and 33% of them consumed it each day [23].

Many factors have been demonstrated to influence children’s food selection and dietary habits, with the most frequent being: place of residence and cultural and socioeconomic factors, including education (parental) and income [24]. In our study, the mother’s level of education was a crucial factor related to the frequency of meat, fish, and egg consumption in children. Other important factors were the place of residence, economic status, and children’s gender. A higher level of mother’s education and family income was positively associated with the frequency of meat and meat product consumption in children. Relationships of food preferences during childhood with parental educational attainment were also observed by others [23,25,26,27]. However, the influence of parental level of education on the children’s consumption of meat products was not the same. The results of the research aiming to determine the impact of parents’ attachment to meat on the meal choices of their children in Germany were contrary to our findings. The study revealed that the higher-educated people belonged to the ‘low meat attachment (low MA)’ group, while the majority of the high MA study population were the moderately educated ones [23]. These data correspond to findings from a nationally representative cross-sectional survey set to analyze red and processed meat consumption in North America. As summarized, in the US and Canada, adults with higher educational attainment were less likely to eat red and processed meat [26]. Results from a Danish cross-sectional study verifying the relationship between education and the intake of red meat also confirm that trend. The better-educated people, both men and women, consumed less red meat and more fruit and vegetables compared to individuals with a low educational level [27]. 

It is worth pointing out that in our population, less than 1% of children avoid eating meat and meat products. Growing health concerns related to red and processed meat consumption and trends towards a more plant-based diet make the number of vegetarians increase. In the European adult population, the prevalence of vegetarianism varies in the range of 2–12% [28], and given the data from a nationally representative study, 3.4% of children and adolescents (6–17 years old) were following a vegetarian diet in Germany [29]. There is evidence that more educated individuals are more likely to follow a plant-based diet [30]. In our study, seven of eight mothers of vegetarian children indicated a middle level of education and one, a higher level. 

In our study, the frequency of egg consumption was surprisingly low. According to the mother’s declaration, only 13.3% of children ate eggs every day or a few times a week. In other investigations performed in Poland, eggs were found to be the most frequently consumed products for breakfast among school-aged children [16]. Likewise, in our study, in this examination, a higher education category and living in the city were linked with a higher egg intake frequency. A similar impact of maternal education level on children’s egg consumption was discovered in studies performed in GB [31]. However, the relationship between education level and egg consumption seems to be different or non-existing in self-serving feeding adults. Data on American adults from the National Health and Nutritional Examination Survey (NHANES III) indicate that respondents more likely to eat eggs had lower education levels [32]. In a Turkish study, an association between egg consumption by adults and their level of education was not observed [33].

The effect of selected factors on the frequency of fish consumption was recorded only in the younger population (children at the age of 7–10 years)—children of better-educated mothers eat fish more often. Our observations are in line with other authors’ reports, indicating that families in which parents have high educational levels consume more healthy foods than in those with a level of education described as low [19,34,35]. There were significant differences in the estimated intake of omega-3 fatty acids (based on fish consumption) by Spanish children and adolescents according to the mother’s educational level, as children/adolescents whose mothers have middle education had a lower intake of DHA + EPA than those with better-educated mothers [36]. Although the effect of family income on the frequency of fish intake was not demonstrated in our study, it was shown that the parental education that affects a child’s dietary behavior is correlated with parents’ socioeconomic status [37]. Thus, some data relate the variation in fish intake to socioeconomic position [7,38,39]; in a Dutch adult population study, only about one-third of the respondents adhered to the fish consumption guidelines, and the group with the lowest income met the fish recommendations less often than respondents with better socioeconomic conditions [7]. According to data from the National Diet and Nutrition Survey (2008–2011), the highest socioeconomic groups were 2.4- to 4-times more likely to consume oily fish compared to UK adults with the lowest socioeconomic status [6]. 

The frequency of milk and dairy product consumption was discussed previously [21]. In the general population, place of residence was the only factor affecting the frequency of dairy product intake, and children living in the countryside ate dairy products more often than their peers from the city, as stated earlier [21]. Here, in our examination, significant cross-associations between the frequency of dairy product, vegetable, and whole-grain consumption were recorded, which might suggest considerable nutritional awareness among mothers of children consuming dairy products regularly [34]. In the younger population, apart from the place of residence, the frequency of dairy consumption was linked to gender and the number of siblings. In our study, girls were more likely to drink milk and eat dairy products. These results differ from the data obtained in the selected nutritional survey of Polish youth [40,41]. In a study examining the frequency of selected milk drink intake among adolescents (N = 830), boys were characterized by a higher frequency of milk consumption than girls [40]. On the other hand, there were no significant relationships between the frequency of milk intake, age, and gender in young people (16–18 years), but statistically, the boys consumed fruit yogurts and curd cheese more often than their female peers [41]. Additionally, in a population of adult Silesians (N = 600, aged 18–78 years), there were no differences in milk intake according to age and gender [15]. Although there is a piece of evidence that a diet rich in animal-based foods (except for fish) is associated with cardiovascular risk factors [42], findings from the CAPS cohort study suggest that a higher dairy intake in early childhood may have a favorable effect on blood pressure in mid-childhood (at the age of 9). The values of systolic and diastolic blood pressure were significantly lower in children in the highest dairy intake quintile compared to those from the lowest quintile [43].

Taken together, our research points to the significant role of maternal education in children’s dietary practices, which might have health implications in the later stages of life.

Our survey has some limitations that must be highlighted. The survey was anonymous and voluntary, and although we obtained information concerning dietary habits from almost 900 respondents, the data were only declarative. Another limitation might be that the statistical test used was not able to detect the confounding effects of other determinants on the frequency of meat and egg consumption; thus, it is not excluded that the effect of some factors may be weaker or nonexistent.

## 5. Conclusions

The present research showed that among the environmental factors surveyed, the mother’s level of education was strongly associated with the frequency of animal-origin food consumption, especially meat and meat products, fish, and eggs. Educational attainment, which affects family consumption patterns, makes the parents of school-age children the main target of health-promoting campaigns to increase nutritional awareness of youth.

## Figures and Tables

**Table 1:** Characteristics of the study population (N = 892) [21].

Environmental Factors		N	%
Place of residence	City	483	54.1
	Village	409	45.9
Mother’s education level	Basic and vocational education	55	6.2
	Secondary education	456	51.1
	Higher education	323	36.2
	Not declared	58	6.5
Mother’s body mass (according to BMI)	Underweight	34	3.8
	Normal body mass	542	60.8
	Overweight	183	20.5
	Obese	45	5.0
	Not declared	88	9.9
Net income per family *	<2000 PLN	200	22.4
	2000–4000 PLN	344	38.6
	>4000 PLN	222	24.9
	not declared	126	14.1
Number of children in the family	1	196	22.0
	2	471	52.8
	3	156	17.5
	4+	69	7.7

* Not all mothers declared income, body mass, and education level. PLN: Polish zloty, the average monthly disposable income per person in 2018 was close to 2,000 PLN; BMI: body mass index.

**Table 2:** The most frequently consumed types of meat (N = 892).

Types of Meat	% of Responses	
	Yes	No
Chicken	96	4
Turkey	24	76
Pork	69	31
Beef	14	86
Others	3	97

Others include veal, rabbit, duck, goose.

**Table 3:** The most frequently consumed meat products (N = 892).

Types of Meat Products	% of responses	
	Yes	No
Ham	82	18
Sausage	42	58
Hotdog	58	42
Pate	22	78
Homemade	10	90
Others	1	99

Others include blood sausage, meat products from horseflesh, and venison.

**Table 4:** Relationships between the frequency of ASF, vegetable, and whole grain consumption.

Foods	Meat	Fish	Eggs	Diary
Vegetables	G = 0.284, *p* < 0.001	G = 0.289, *p* < 0.001	G = 0.203, *p* < 0.001	G = 0.097, *p* < 0.05
Grains	NS	G = 0.132, *p* < 0.01	G = 0.204, *p* < 0.001	G = 0.174, *p* < 0.001

NS—not statistically significant.

## Data Availability

Not applicable.
